# ASPP2 suppresses tumour growth and stemness characteristics in HCC by inhibiting Warburg effect via WNT/β‐catenin/HK2 axis

**DOI:** 10.1111/jcmm.17687

**Published:** 2023-02-08

**Authors:** Beibei Liang, Yuan Jiang, Shaohua Song, Wei Jing, Hao Yang, Li Zhao, Ya Chen, Qiqi Tang, Xuhui Li, Lisha Zhang, Haili Bao, Gang Huang, Jian Zhao

**Affiliations:** ^1^ Shanghai Key Laboratory of Molecular Imaging Jiading District Central Hospital Affiliated Shanghai University of Medicine and Health Sciences Shanghai China; ^2^ Medical Engineering Department The Affiliated Hospital of QingDao University Qingdao Shandong China; ^3^ Liver Transplantation Center, Department of General Surgery, Ruijin Hospital Shanghai Jiaotong University School of Medicine Shanghai China; ^4^ Department of Surgery, Changhai Hospital Navy Military Medical University Shanghai China; ^5^ Department of Nuclear Medicine, Institute of Clinical Nuclear Medicine, Renji Hospital, School of Medicine Shanghai Jiao Tong University Shanghai China; ^6^ Shanghai Skin Disease Hospital, School of Medicine Tongji University Shanghai China; ^7^ Department of Organ Transplantation, Shanghai Changzheng Hospital Navy Military Medical University Shanghai China

**Keywords:** ASPP2, hepatocellular carcinoma, tumour growth, Warburg effect

## Abstract

Abnormal energy metabolism is one of the characteristics of tumours. In the last few years, more and more attention is being paid to the role and regulation of tumour aerobic glycolysis. Cancer cells display enhanced aerobic glycolysis, also known as the Warburg effect, whereby tumour cells absorb glucose to produce a large amount of lactic acid and energy under aerobic conditions to favour tumour proliferation and metastasis. In this study, we report that the haploinsufficient tumour suppressor ASPP2, can inhibit HCC growth and stemness characteristics by regulating the Warburg effect through the WNT/β‐catenin pathway. we performed glucose uptake, lactate production, pyruvate production, ECAR and OCR assays to verify ASPP2 can inhibit glycolysis in HCC cells. The expression of ASPP2 and HK2 was significantly inversely correlated in 80 HCC tissues. Our study reveals downregulation of ASPP2 can promote the aerobic glycolysis metabolism pathway, increasing HCC proliferation, glycolysis metabolism, stemness and drug resistance. This ASPP2‐induced inhibition of glycolysis metabolism depends on the WNT/β‐catenin pathway. ASPP2‐regulated Warburg effect is associated with tumour progression and provides prognostic value. and suggest that may be promising as a new therapeutic strategy in HCC.

## 1 INTRODUCTION

The most common primary malignancy in liver is hepatocellular carcinoma (HCC) and one of the leading causes of tumour‐related death in the world.^1^ The growth and development of liver cancer is a complex process with many risk factors and progression steps. In clinical practice, there are problems in the treatment of HCC, such as incomplete treatment, high recurrence rate and easy drug resistance. Therefore, finding the intervention target of metastasis, recurrence and drug resistance is of great significance to the treatment of HCC.

Reprogramming of energy metabolism is one of the top ten features of cancer progressions. Increasing research suggest that altered energy metabolism play an important role in cancer.^2^ Cancer cells often display enhanced aerobic glycolysis, also known as the Warburg effect, whereby tumour cells absorb glucose to produce a large amount of lactic acid and energy under aerobic conditions, to favour tumour proliferation and metastasis. It has been reported that enhanced aerobic glycolysis is partly due to changes in metabolic kinases in the metabolic pathway, including hexokinase 2 (HK2), pyruvate kinase M2 subtype (PKM2), lactate dehydrogenase A (LDHA), etc.^3^, ^4^ The scientific evidence reveals that enhanced aerobic glycolysis is driven by oncogenic signalling, such as MAPK, PI3K/AKT,^5^ and WNT/β‐catenin signalling.^6^, ^7^ However, the role and molecular mechanism of aerobic glycolysis in the development of HCC are still not clear.

ASPP2 is a member of the ankyrin‐repeat, SH3‐domain and proline‐rich region‐containing protein (ASPP) family and is a haploinsufficient tumour suppressor.^8^, ^9^ Aberrant expression of has been found in various human cancers.^10^ Our previous study found that ASPP2 is downregulated by DNA methylation in HCC.^11^ ASPP2 inhibits tumour growth and metastasis through the regulation of apoptosis, autophagy and epithelial plasticity.^8^, ^12^, ^13^, ^14^ Recently, we have found that the downregulation of ASPP2 resulted in higher cholesterol levels in liver cancer cells, and contributed to tumour stem cell‐like properties and tumour growth.^15^ Altogether these findings indicate that the abnormal expression of ASPP2 plays an important role in the occurrence and development of HCC.

In the study of the relationship between ASPP2 and metabolism, we found that glucose metabolism was closely related to ASPP2 level in HCC. Our GC–MS data showed that after interfering with LV‐shAspp2, enhancement of the Warburg effect; cell glucose level, pyruvate and lactic acid all increased. This was supported by our glucose ^18^F‐FDG uptake experiment, which also showed significantly increased glucose uptake in the ASPP2 interference group. We further demonstrated that ASPP2 reduces the ability of tumour‐initiating capabilities and tumour growth by inhibiting the aerobic glycolysis metabolism in HCC.

## 2 MATERIALS AND METHODS

### 2.1 Cell lines and culture conditions

Hep‐G2 (PRID:CVCL_0027) cell were purchased from Procell Life Science& Technology Co. Ltd.; Huh‐7 (PRID: CVCL_0336), Hep‐3B (PRID: CVCL_0326) and HCC‐LM3 (PRID:CVCL_4972) cell were purchased from Hunan Fenghui Biotechnology Co., Ltd. All these cell lines were cultured in DMEM (Gibco) supplemented with 10% (vol/vol) FBS (Gibco), 100 U/mL penicillin and 100 μg/mL streptomycin (Invitrogen) at 37°C in a humidified incubator containing 5% CO2. All experiments were performed with mycoplasma‐free cells.

### 2.2 Western blotting

Total protein was extracted from cell samples with Cell lysis buffer for Western and IP (Beyotime) with 1% PMSF (Beyotime). The protein concentrations were measured using the Bicinchoninic Acid (BCA) kit (YEASEN). Proteins were separated by SDS‐PAGE and transferred to PVDF membrane (Millipore). Then the blots were detected with super ECL Detection reagent (YEASEN) using Bio‐Rad ChemiDocTM (Azure Biosystems) after incubated with primary antibodies overnight at 4°C and probed with secondary antibodies at room temperature for 1–2 h. The detail antibodies information can be found in Table S1. XAV‐939 (HY‐15147), ICG‐001 (HY‐14428) were all purchased from MedChemExpress, 2‐DG (SIGMA, D8375), 3‐bp (S5426) was purchased from Selleck. Other details can be found in the Appendix S1.

### 2.3 Real‐time PCR

We used the RNA Miniprep Kits (QIAGEN) to isolate total cellular RNA, according to the manufacturer's instruction, and then reverse transcribed in to cDNA, and the thermal cycling settings were as follows: 95°C for 30 s, then 40 cycles of 95°C for 10 s and 60°C for 30 s. Relative mRNA levels were determined using the 2(−∆Ct) (∆Ct = Ct target ‐ Ctβ‐actin) method and CB was used as an endogenous control. The primer sequences are listed in Table S2. The experiments have been repeated at least three times.

### 2.4 Lentivirus shRNA production

We designed three pairs of cDNA oligonucleotides targeting ASPP2 mRNA expression, using web‐based software from Invitrogen and InvivoGen Inc. After synthesis, we inserted these double‐strand oligos into the vector pENTR/U6 (Invitrogen) and sequenced the resulting plasmids to ensure the shRNA construct targeted human ASPP2 expression or were scrambled, which were generated and designated as LV‐shAspp2 and LV‐shNon. Then, the plasmids were transfected into HCC cells and gene silencing efficiency was validated 72 h after transfection by real‐time PCR and Western blot (Figure S1).Other details about Lentivirus shRNA productions can be found in the Appendix S1.

### 2.5 Glucose uptake and lactate /pyruvic production

Tumour cells (1 × 10^6^) were seeded into 6‐well plate culture for 24 h after cells were adhered. For measurement of glucose, lactate and pyruvic concentration, the culture medium was replaced with serum‐free medium and 10 μM XAV‐939 (MCE, HY‐15147) or 10 μM ICG‐001 (MCE, HY‐14428) for 24 h and then the culture medium was collected. The glucose uptake assay was performed using a glucose assay kit (Sigma‐Aldrich, GAGD20), and glucose consumption was calculated by deducting the measured glucose concentration in the medium from the original glucose concentration. Lactate production assay was performed using Lactate Assay kit (Jiancheng Bioengineering, AD19‐2‐1) according to the manufacturer's protocol. Pyruvic production assay was performed using Pyruvic Assay kit (Solarbio, BC0540). Results were normalized according to cell numbers. Details can be found in the Appendix S1.

### 2.6 Cellular glycolysis stress and oxygen consumption assay

Cellular oxidative phosphorylation and glycolysis alternations were determined with the Seahorse XF24 Flux Analyser (Seahorse Bioscience) by measuring extracellular acidification rates (ECAR) and oxygen consumption rates (OCR), respectively, in real time according to the manufacturer's instructions. HCC‐LM3 were seeded in a XF24‐well plate (Seahorse Bioscience) at a density of 3 × 10^5^ per well with ASPP2 knockdown cells and control cells, then allowed to attach overnight. OCR was assessed using sequential injection of 1 μM oligomycin, 1 μM carbonyl cyanide 4‐(trifluoromethoxy) phenylhydrazone (FCCP), 1 μM antimycin and rotenone. For assessment of ECAR, cells were incubated with unbuffered medium followed by injection of 10 mM glucose, 1 μM oligomycin (Sigma‐Aldrich) and 80 mM 2‐deoxyglucose (Sigma‐Aldrich). The basal levels of OCR and ECAR were recorded first, then the OCR and ECAR levels were recorded after sequential injection of the compounds that inhibit the respiratory mitochondrial election transport chain, ATP synthesis or glycolysis. Both OCR and ECAR measurements were normalized to cell numbers and reported as pmoles/min for OCR and mpH/min for ECAR.

### 2.7 Animal studies and image

Four‐week‐old male athymic BALB/c nude mice were purchased from the Shanghai Experimental Animal Center of Chinese Academic of Sciences (Shanghai, China) and were maintained in specific pathogen‐free conditions. Animal care and experimental protocols were conducted in accordance with the guidelines of Shanghai Medical Experimental Animal Care Commission. For in vivo treatment, HCC‐LM3 cells (1 × 10^7^) infected with shNon‐Luc and shAspp2‐Luc (at a MOI of 50) were implanted subcutaneously into the flank of nude mice (five in each group, male BALB/c nu/nu). By day 7, tumours were well established in the mice with an average size of ~300 mm^3^. Mice were intraperitoneally given 2‐DG (500 mg/kg diluted in isotonic saline) daily for 3 days. Glucose uptake in the tumour xenografts was monitored by ^18^F‐FDG micro‐PET/CT. The tumours were also scanned using PET/CT scanner (Shanghai) after injecting them intravenously with 100 μL saline containing 100 μCi ^18^F‐FDG. The standardized uptake value of the region of interest was then evaluated manually. And, whole body bioluminescent images of HCC‐LM3‐Luc tumour xenograft mice were determined by the Xenogen IVIS 100 imaging System (PEIVIS Spectrum). At the end of the experiment, the mice were sacrificed and tumours were photographed and weighed. More details about tumour xenograft model can be found in the Appendix S1.

### 2.8 Cell viability and chemo‐resistance assays

Cells infected with shAspp2 and shNon were seeded into 96‐well plates (10,000 cells/well).Then cells were treated with 60 ug/mL 2‐DG (Sigma), 10 ug/mL 5‐FU (Selleck), and 20 ug/mL Oxaliplatin (Sanofi) for 24, 48 h. Cell viability was measured by CCK‐8 assay reagent (Beyotime). The experiments have been repeated at least three times.

### 2.9 Immunohistochemical staining

The expressions of ASPP2, HK2 and PKM2 were analysed with ImageScope system in formalin‐fixed, paraffin‐embedded sections of primary tumours. Other details can be found in the Appendix S1.

### 2.10 Patient samples

We performed a tissue microarray constructed by Shanghai Weiao Biotechnology Co., Ltd, China (Weiao Biotechnology Co., ZL‐LVC1605). Weiao Biotechnology Co. was responsible for obtaining informed consent from all subjects. Eighty HCC tumour tissue samples made for microarray were obtained from patients who had undergone curative hepatic resection between 2008 and 2015. Patient's samples were approved to use for research purposes by medical Ethics Committee of Shanghai University of Medicine & Health Sciences (2018‐GZR‐18‐310110196803058627). The clinicopathologic features of the patients were summarized in Table 2. Other details about patient samples can be found in the Appendix S1.

### 2.11 Statistical analysis

The Kaplan–Meier survival analysis was performed to compare patient survival data. The Student's *t*‐test was used to compare data between two groups. Differences were considered statistically significant at *p* < 0.05.

## 3 RESULTS

### 3.1 Downregulation of ASPP2 enhances the Warburg effect in HCC cells

**FIGURE 1:**
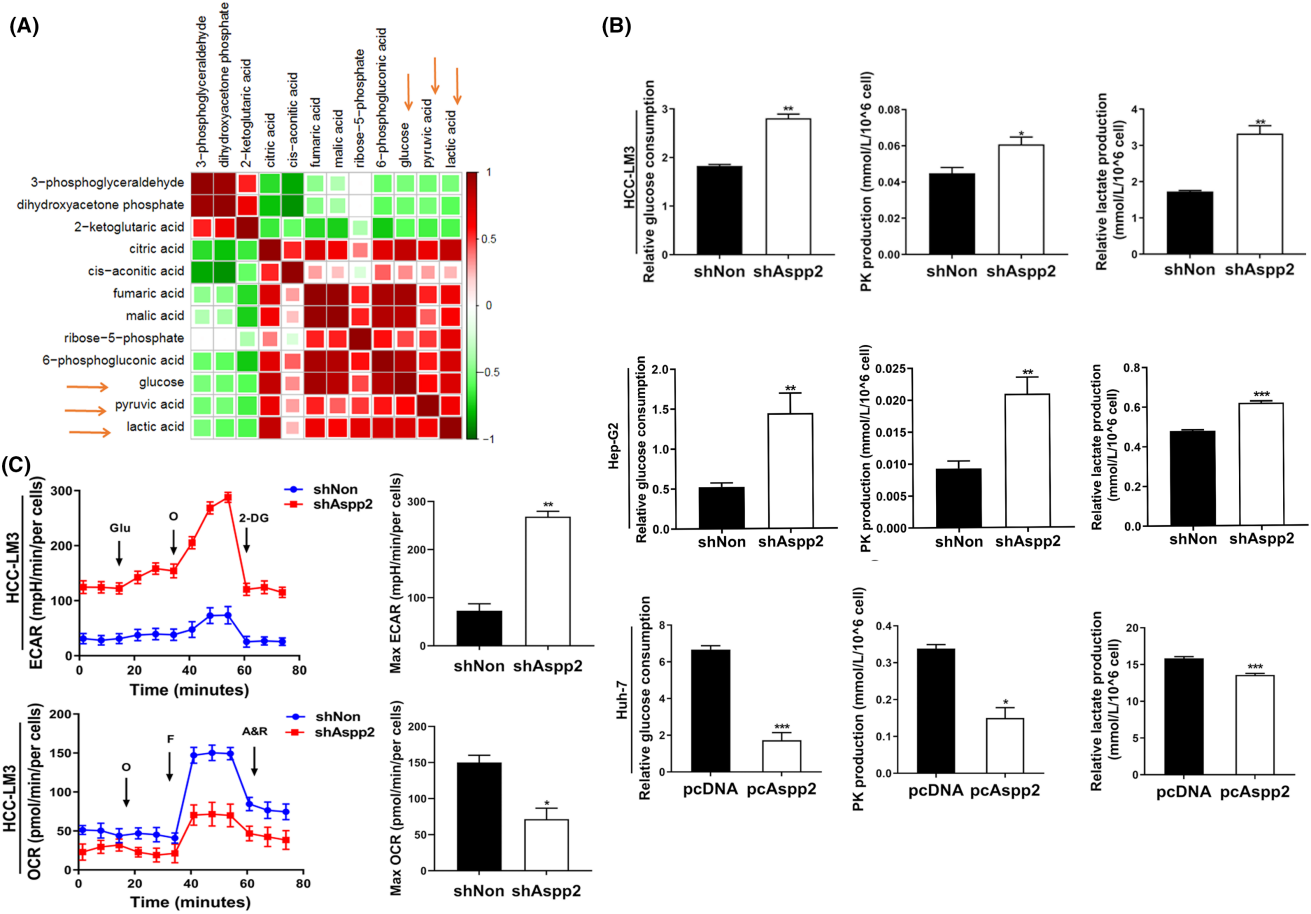
Downregulation of ASPP2 enhance tumour aerobic glycolysis in HCC cells. (A) Non‐targeted metabolomics were measured by GC–MS methods, using the internal standard strategy as described in methods. Correlation matrix analysis show changes in metabolomics between HCC‐LM3 infected with LV‐shAspp2 and LV‐shNon. Arrows illustrate pyruvic acid, lactate acid and glucose were significant correlation in HCC‐LM3 infected with LV‐shAspp2 and LV‐shNon. (B) Pyruvic acid production, lactate production and glucose consumption in HCC‐LM3 and Hep‐G2 infected with LV‐shAspp2 or LV‐shNon and Huh‐7 cells transfected with ASPP2 plasmid or control. (C) Effects of ASPP2 on oxygen consumption ratio (OCR) and extracellular acid ratio (ECAR) in HCC‐LM3. O, oligomycin; F, FCCP (carbonyl cyanide 4‐[trifluoromethoxy] phenylhydrazone); A&R, antimycin A and rotenone; Glu, glucose; O, oligomycin; 2‐DG, 2‐deoxyglucose. All data are shown as the mean ± SEM of 3 independent experiments. *p < 0.05; **p < 0.01; ***p < 0.001

We carried out metabolomics analysis in HCC‐LM3 cells infected with LV‐shAspp2 and LV‐shNon using gas chromatography mass spectrometry (GC–MS). GC–MS analysis revealed significant increases in glucose, pyruvic acid and lactic acid in HCC‐LM3 cells with downregulated ASPP2 (Table 1). Obvious correlation were observed in metabolites glucose, pyruvic acid and lactic acid in LV‐shAspp2 group and LV‐shNon group by correlation matrix analysis (Figure 1A). ASPP2 knockdown greatly stimulated the Warburg effects in HCC cells. Much higher levels of glucose uptake, pyruvic acid and lactate production were observed in HCC‐LM3 and Hep‐G2 cells with ASPP2 knockdown, and lower levels of glucose uptake, pyruvic acid and lactate production were observed in Huh‐7 cells with ASPP2 overexpression (Figure 1B). We further compared several key cellular metabolic and bioenergetic indicators between HCC‐LM3 cells with ASPP2 knockdown and control cells, respectively. Results showed that ASPP2 knockdown greatly increased extracellular acidification rates (ECAR) and decreased basal and maximal oxygen consumption rates (OCR) in HCC‐LM3 cells (Figure 1C).

**TABLE 1:** Gas chromatography/Mass spectrometry analysis (GC–MS)

Metabolites	*p* (*t*‐test)	FC (shAspp2/shNon)
Glucose*	1.19 E‐02*	0.99*
DHAP (dihydroxyacetone phosphate)	1.55 E‐02	−1.65
3‐phosphoglyceraldehyde	2.62 E‐02	−2.20
Pyruvic acid*	4.9 E‐02*	1.024*
Lactic acid*	3.88 E‐02*	0.70*
Ribose‐5‐phosphate	1.12 E‐01	0.60
Glucose‐6‐phosphate	2.76 E‐01	−1.25
6‐phosphogluconic acid	2.00 E‐02	3.41

*Note*: The *p*‐value was calculated from Student's *t*‐test. FC, fold change, was calculated as a binary logarithm of the average mass response (normalized peak area) ratio between Group shAspp2 vs Group shNon, where a positive value means that the average mass response of the metabolite in Group shAspp2 is larger than that in Group shNon.

* *P* < 0.05.

**FIGURE 2:**
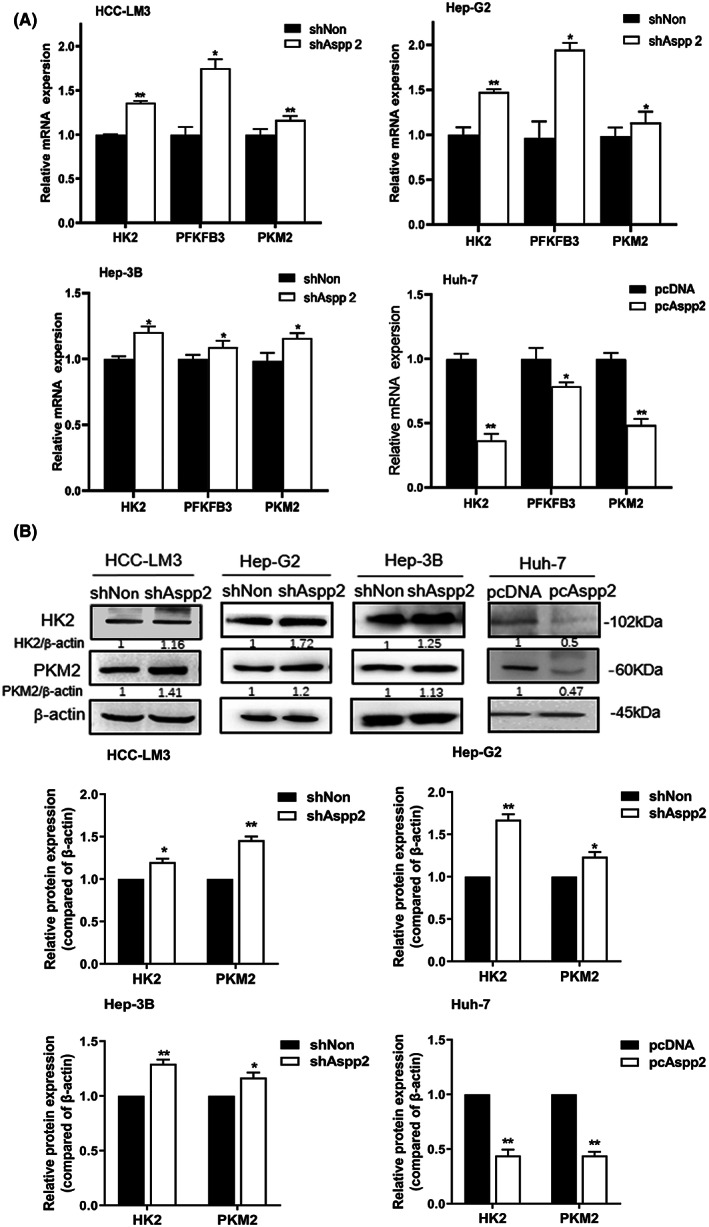
ASPP2 Decreases the expression of glycolytic enzymes in HCC cells. (A) The mRNA levels of HK2, PFKFB3 and PKM2 were determined after ASPP2 knockdown in HCC‐LM3, Hep‐G2 and Hep‐3B cells. The mRNA levels of HK2, PFKFB3 and PKM2 were determined after ASPP2 overexpression in Huh‐7 cell. (B) The protein levels of HK2 and PKM2 were determined after ASPP2 knockdown in HCC‐LM3, Hep‐G2 and Hep‐3B cells, as well as in Huh‐7 cells with overexpressed‐ASPP2. The protein expression had been quantified and presented in a bar graph. The same bands of β‐Actin in Hep‐3B cells are used in (B) and Figure S1C. And, the same bands of β‐Actin in Huh‐7 cells are used in (B) and Figure S1D. All data are shown as the mean ± SEM of 3 independent experiments. *p < 0.05; **p < 0.01

To further investigate the mechanism of ASPP2 in the regulation of aerobic glycolysis, we assessed the effect of ASPP2 on the expression of key glycolytic kinases. We measured the mRNA levels of glycolytic enzymes using real‐time PCR and found that the expression levels of most enzymes were changed (Figure S2). The mRNA level of key glycolytic regulators like hexokinase 2 (HK2), 6‐phosphofructo‐ 2‐kinase /fructose‐2, 6‐bisphosphatase 3 (PFKFB3) and pyruvate kinase M2 (PKM2) were notably elevated in ASPP2‐depleted HCC‐LM3, HepG2 and Hep3B cells, whereas these were decreased in ASPP2‐overexpressed Huh‐7 cells (Figure 2A). We also examined the protein levels of HK2 and PKM2 after ASPP2 overexpression or knockdown in HCC cells, and found that the changes were consistent with that in mRNA levels (Figure 2B). Taken together, we found that downregulation of ASPP2 promoted aerobic glycolysis and the expression of key glycolytic kinases in HCC cells.

In addition, ASPP2 is identified as an activator of the p53 family. In order to validate the role of p53 in ASPP2‐regulated aerobic glycolysis, we used siRNA (siP53, GenePharma, Shanghai) to deplete p53 expression in HCC‐LM3 cells, which harbouring wild type p53 and ASPP2.^11^ As shown in Figure S3, we found the depletion of p53 did not change the expression of HK2, PKM2 and PFKFB3, as well as glucose consumption, lactate and PK production in HCC‐LM3 cells. Thus, ASPP2 may regulate aerobic glycolysis in a p53‐independent manner.

### 3.2 ASPP2 modulates the Warburg effect by regulating β‐catenin level

We then investigated the mechanism of Warburg effect inhibition by ASPP2 in HCC.

Previously, it has been demonstrated that WNT/β‐catenin signalling promotes aerobic glycolysis directly or through its target gene c‐myc in cancer cells.^16^ MYC is a ‘master regulator’ of glycolysis and tumour proliferation, which can transcriptionally upregulate several key genes involved in aerobic glycolysis like HK2 and PKM2.

**FIGURE 3:**
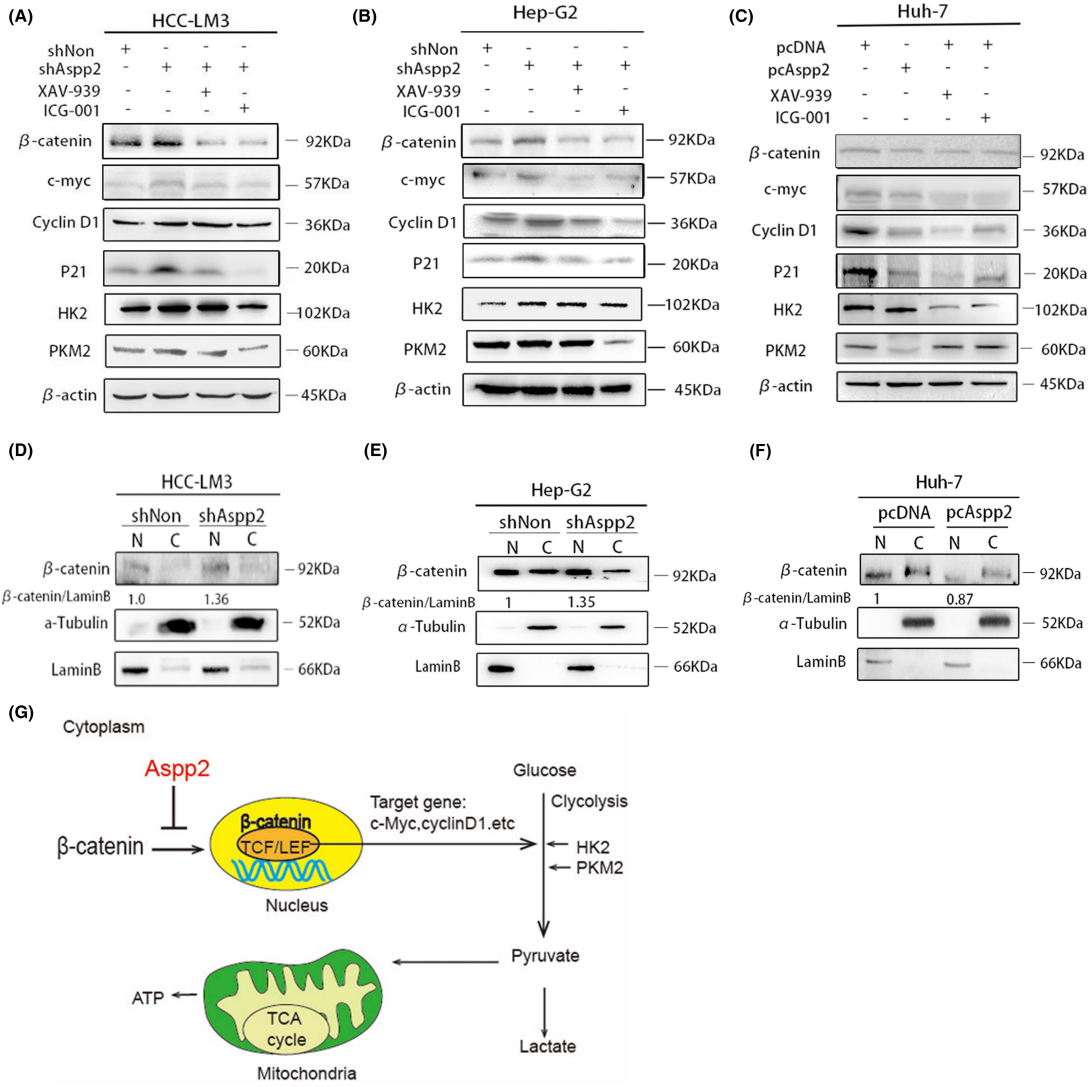
ASPP2 depresses β‐catenin and its downstream target expression. After treatment with XAV‐939 (10 μM) or ICG‐001 (10 μM) for 24 hours, the protein level of β‐catenin and its downstream target were analysed using Western blot in (A) HCC‐LM3 cells and (B) Hep‐G2 cells with ASPP2 knockdown, as well as in (C) Huh‐7 cells with ASPP2 overexpression, compared to relative control cells. The subcellular distribution of β‐catenin in cytoplasm (marked as C) and nucleus (marked as N) of HCC‐LM3 cells (D) and Hep‐G2 cells (E) after ASPP2 knockdown, as well as (F) Huh‐7 cells with ASPP2 overexpression. α‐tubulin and Lamin B were served as internal control of cell cytoplasm and nucleus accordingly. (G) Schematic representation for the mechanism of ASPP2 mediated glycolysis in HCC. Schematic illustrates how ASPP2 inhibit β‐catenin and its downstream target expression, c‐myc, CyclinD1 and P21, as well as glycolytic key enzymes HK2 and PKM2, which reduce the production of lactate acid and pyruvic acid in glycolysis

Accordingly, we analysed the levels of β‐catenin and its downstream targets, c‐myc, CyclinD1 and P21, as well as glycolysis kinases HK2 and PKM2, with and without WNT/β‐catenin pathway inhibitors XAV‐939 or ICG‐001 in ASPP2 different expression groups. Our results show that the expression of β‐catenin, c‐myc, CyclinD1, P21, HK2 and PKM2 were elevated in ASPP2‐depleted HCC‐LM3 and Hep‐G2 cells (Figure 3A,B), but was decreased in ASPP2‐overexpressed Huh‐7 cells (Figure 3C). XAV‐939 is a potent tankyrase inhibitor that targets Wnt/β‐catenin signalling. ICG‐001 can also antagonize Wnt/β‐catenin/TCF‐mediated transcription. Both greatly attenuated ASPP2‐depletion‐induced increase of HK2 and PKM2 (Figure 3A,B). We also found that the nuclear β‐catenin were increased in ASPP2‐ knockdown HCC‐LM3 and Hep‐G2 cells, but was decreased in ASPP2‐overexpressed Huh‐7 cells (Figure 3D–F). Schematic representation for ASPP2 mediated aerobic glycolysis in HCC is shown (Figure 3G).These results illustrate that interference of ASPP2 can promote β‐catenin from the cytoplasm into the nucleus and effect its downstream targets and glycolysis enzymes in HCC cells.

**FIGURE 4:**
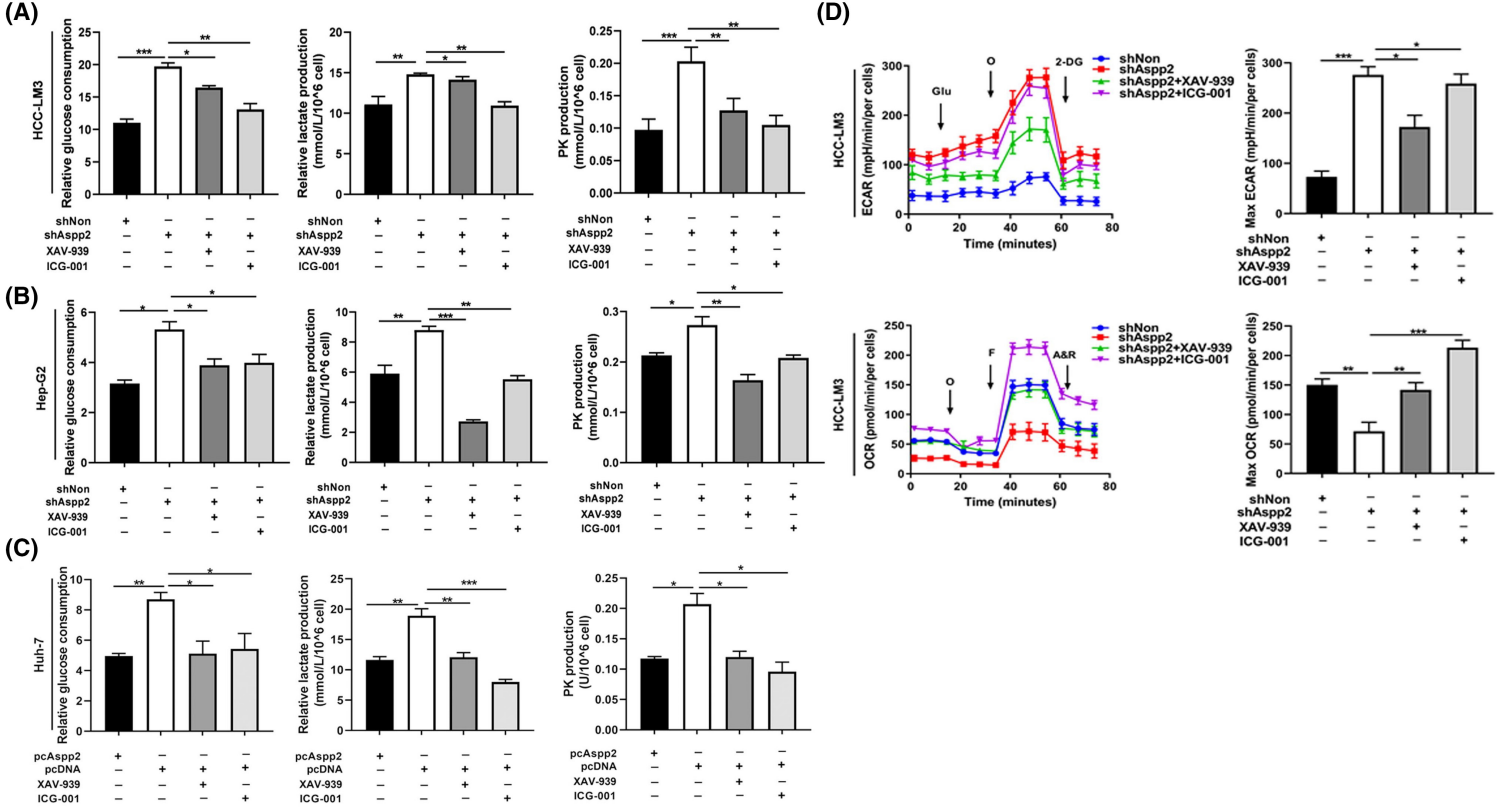
ASPP2 reduces the Warburg effect by inhibition of β‐catenin signalling. (A–C) Glucose, lactate and pyruvic acid (PK) production in HCC‐LM3, Hep‐G2 cells infected with LV‐shAspp2 or LV‐shNon and Huh‐7 cells with ASPP2 overexpression, after treatment with XAV‐939 (10 μM) or ICG‐001 (10 μM) for 24 h. (D) HCC‐LM3 cells infected with LV‐shAspp2 or LV‐shNon, after treatment with XAV‐939 (10 μM) or ICG‐001 (10 μM) for 24 h. Effects of ASPP2 on oxygen consumption ratio (OCR) and extracellular acid ratio (ECAR). All data are shown as the mean ± SEM of 3 independent experiments. *p < 0.05; **p < 0.01; ***p < 0.001

We next investigated the role of β‐catenin in the ASPP2‐dependent Warburg effect. Our results indicate that β‐catenin inhibited by XAV‐939 or ICG‐001 greatly abolished the promotion effects of ASPP2‐depletion on the Warburg effect as indicated by decreased glucose consumption, pyruvic acid and lactate production in HCC cells (Figure 4A–C). Further, both XAV‐939 and ICG‐001 abolished the enhanced level of extracellular acidification rates (ECAR) and promoted the level of oxygen consumption rates (OCR) in ASPP2‐knockdown HCC‐LM3 cells (Figure 4D). These data suggest that ASPP2‐depletion‐induced Warburg effect is promoted by the activation of WNT/β‐catenin pathway in HCC cells.

### 3.3 Warburg effect is essential for maintaining tumour‐initiating capability and tumour‐stemness characteristics in ASPP2‐depleted HCC cells

**FIGURE 5:**
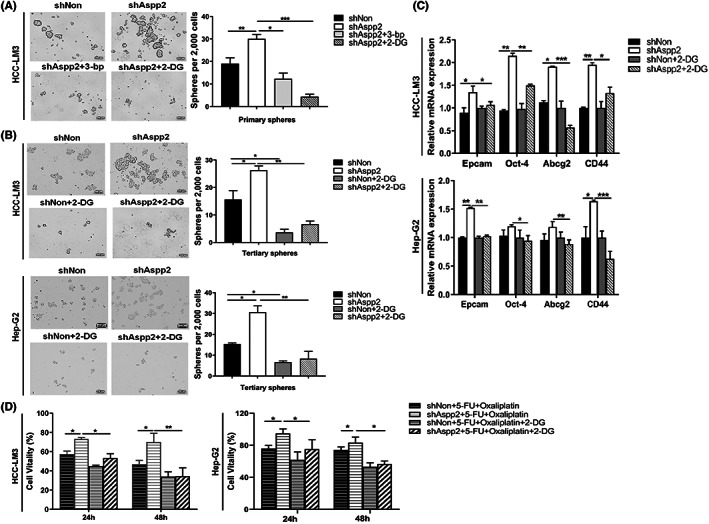
Warburg effect is essential for maintaining tumour‐initiating capability in ASPP2‐depleted HCC cells. (A) Left: Representative images of the first spheroid formation in HCC‐LM3 cells with 3‐bp (60 μM) or 2‐DG (0.1 μM) after knocking‐down of ASPP2. Right: the number of spheres formation per 2000 cells. Scale bar, 100 μm. (B) Left: Representative images of the tertiary spheres formation in HCC‐LM3 and Hep‐G2 cells with 2‐DG (0.1 μM) after knocking‐down of ASPP2. Right: the number of spheres formation per 2000 cells. Scale bar, 100 μm. (D) qRT‐PCR gene expression of Epcam, Oct‐4, Abcg2 and CD44 in HCC‐LM3 and HepG2 cells transfected with the indicated lentivirus and treated with 2‐DG (0.1 μM). (D) Cell viability of infected HCC‐LM3 and Hep‐G2 cell treated with 5‐FU (10 ug/mL) and Oxaliplatin (1 μM) with or without 2‐DG (0.1 μM) was analysed by CCK‐8 assay in 24, 48 h

To verify the importance of the Warburg effect in ASPP2‐depletion‐induced tumour enhancement in HCC cells, we inhibited the Warburg effect with 2‐DG and 3‐bp, inhibitors of glycolysis that can induce cancer cell death by depriving intracellular glucose levels. In a suspension culture system, the numbers and the size of cell primary spheres and tertiary spheres were greatly decreased in 2‐DG or 3‐bp treated ASPP2‐depleted HCC‐LM3 and Hep‐G2 cells (Figure 5A,B). In addition, we assessed the expression of stemness‐associated genes by qRT‐PCR. ASPP2 depletion resulted in the upregulation of transcription factor Oct‐4, ATP‐binding cassette transporter (Abcg2), epithelial cell adhesion molecule Epcam and CD44, in HCC‐LM3 and Hep‐G2 cells. These alterations were compromised by 2‐DG treatment (Figure 5C). Resistance to chemotherapy is regarded as another property of tumour‐initiating cells. Compared to control cells, ASPP2‐depleted HCC‐LM3 and Hep‐G2 cells displayed significantly higher resistance to 5‐FU and Oxaliplatin co‐treatment. However, this resistance was attenuated with 2‐DG (Figure 5D). These results suggested that Warburg effect is critical for maintaining tumour‐initiating capability and tumour‐stemness characteristics, which contributes to drug resistance in ASPP2‐depleted HCC cells.

### 3.4 ASPP2 depletion‐induced tumour growth is related with glucose uptake in vivo

**FIGURE 6:**
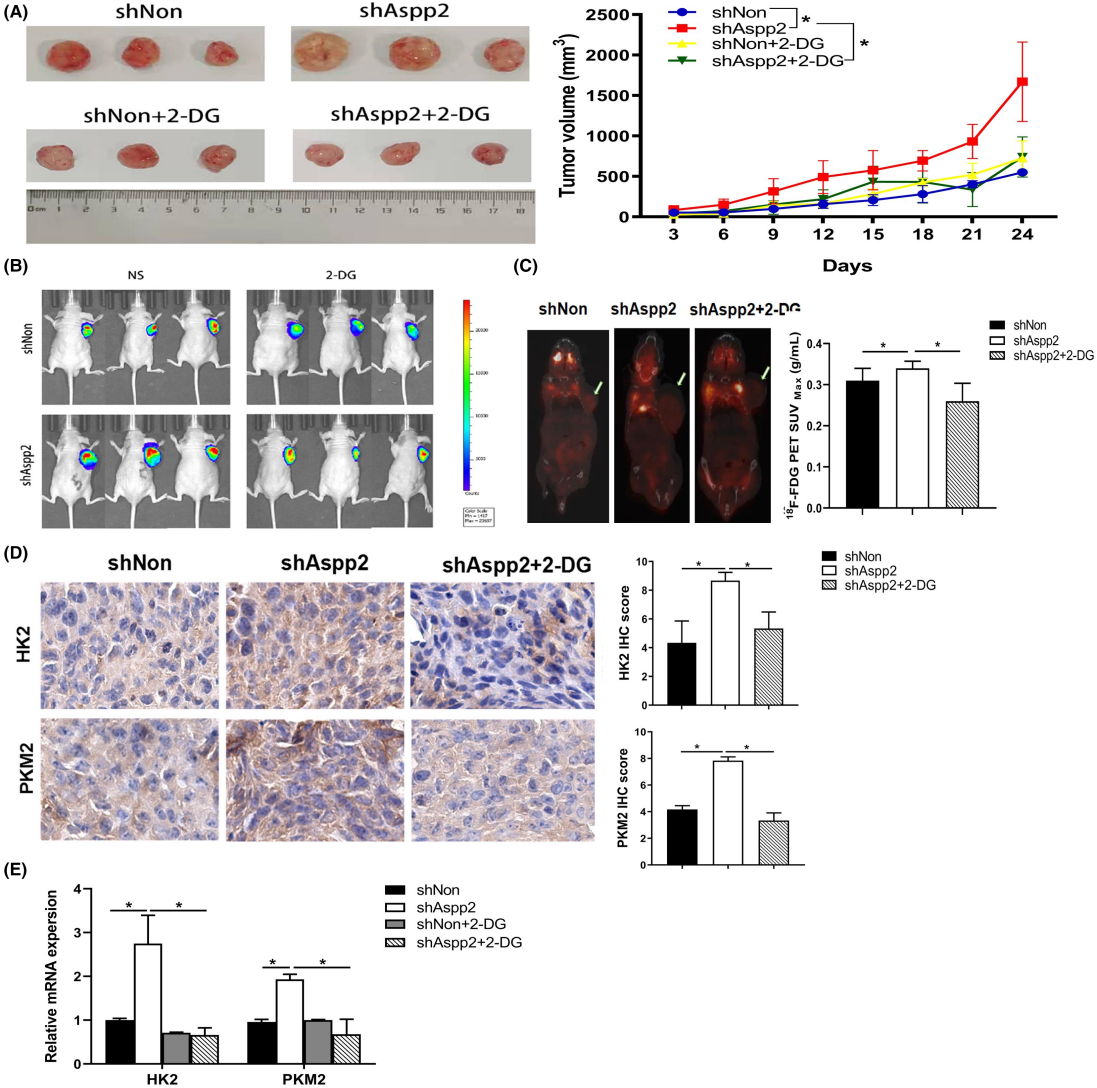
Downregulation of ASPP2 promoted tumour growth in vivo by activating Warburg effect. (A) Representative dissected tumours from nude mice treated with physiological saline or with 2‐DG (500 mg/kg) for 3 weeks (left) and corresponding volume measurement (right), asterisk (*) indicates *p* < 0.05. (B) Whole body bioluminescent images of representative HCC‐LM3‐luc tumour xenograft mice were determined by the Xenogen IVIS 100 imaging System. (C) Representative micro‐PET/CT images of xenograft tumours induced in nude mice by LV‐shAspp2 or LV‐shNon HCC‐LM3 cells, with the arrow representing the tumour site. (D) HK2 and PKM2 were detected by immunohistochemical staining in tumour xenograft mice tissue. Representative pictures are shown (Scale bars: 100 μm). (E) The mRNA of HK2 and PKM2 were examined by q‐PCR in mice tumour tissues in different treated group randomly, values are given as the means ± SD of 3 mice

To further confirm that the Warburg effect contributes to tumour growth in ASPP2‐depletion HCC cells, HCC‐LM3 cells expressing shAspp2‐Luc or shNon‐Luc were injected into the flank of nude mice, treated with or without 2‐DG. Twenty one days after xenograft, 2‐DG treatment significantly delayed the growth rate and reduced the tumour volume in ASPP2‐silenced HCC‐LM3 xenografts (Figure 6A). Furthermore, the results of in vivo imaging showed that 2‐DG treatment blocked ASPP2 depletion‐induced tumour growth in HCC‐LM3 xenograft models (Figure 6B). The ^18^F‐FDG position emission tomography‐computed tomography (PET/CT) showed ASPP2‐depletion significantly induced ^18^F‐FDG uptake in tumours, which was attenuated by 2‐DG treatment (Figure 6C). The glycolytic key enzymes of HK2 and PKM2 were found highly expressed in ASPP2‐depleted tissues from xenografts and were decreased by 2‐DG treatment (Figure 6D). Additionally, the mRNA level of HK2 and PKM2 were increased in the ASPP2‐depletion group and was attenuated by 2‐DG treatment (Figure 6E). These results demonstrate that ASPP2 depletion‐induced tumour growth is related to glucose uptake and the Warburg effect.

### 3.5 ASPP2‐regulated Warburg effect is associated with tumour progression and provides prognostic value

**FIGURE 7:**
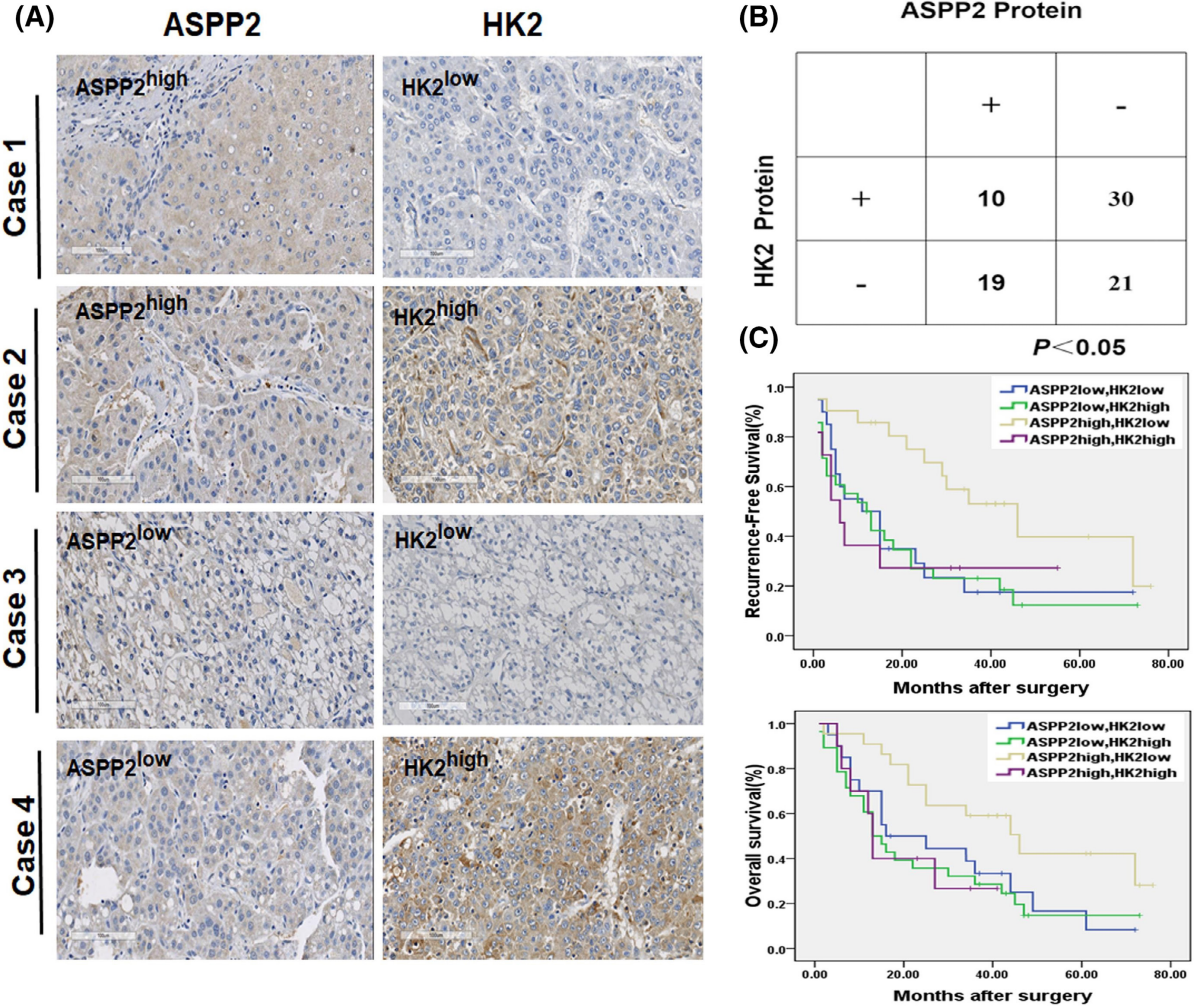
Expression of ASPP2 correlates negatively with HK2 in surgical specimens of HCC. (A) ASPP2 and HK2 were detected by immunohistochemical staining in 80 HCC samples. Representative pictures are shown for four patients. (Scale bars: 100 μm). (B) Table of ASPP2 and HK2 high and low expression according to immunohistochemistry scoring. Significant negative correlation between ASPP2 and HK2 expression was found, Fisher's test P < 0.05. (C) Top: Overall survival (OS) and Bottom: recurrence‐free survival (RFS) rate curves of the analysed four subgroups (ASPP2 low/HK2 low; ASPP2 low/HK2 high; ASPP2 high /HK2 low; ASPP2 high/HK2 high), (*n* = 80). Kaplan–Meier analysis showed RFS (*p* < 0.05) and OS (*p* < 0.05) were significantly best amongst patients with ASPP2‐high and HK2‐low expression

To assess the clinical relevance of ASPP2‐regulated Warburg effect, we further examined ASPP2 and HK2 protein expression in 80 HCC tissues by immunohistochemistry. 38% (30/80) cases showed low ASPP2 expression and high expression of HK2, whilst 23% (19/80) cases showed high ASPP2 expression and low HK2 expression. Thus, the expression of ASPP2 and HK2 was significantly inversely correlated (*p* < 0.05; Figure 7A,B).

The relationships between ASPP2 and HK2 expression and clinical features were statistically analysed in Table 2. A significant correlation between HK2, ASPP2 expression and tumour volume was observed (*p* = 0.027): patients with ASPP2‐low and HK2‐high expression were prone to having a larger tumour volume. Further, patients with ASPP2‐high and HK2‐low expression had the longest recurrence time (*p* = 0.037). Consistent with these findings, patients with ASPP2‐high and HK2‐low expression exhibited the best recurrence‐free survival (RFS), *p* = 0.039, as well as overall survival (OS), *p* = 0.047 (Figure 7C). These data suggest that ASPP2‐regulated Warburg effect contributes to tumour progression in HCC patients.

**TABLE 2:** The associations of ASPP2 and HK2 expression with clinicopathologic characteristics in 80 patients with HCC

	Whole study (*n* = 80)			ASPP2 negative group (*n* = 51)			ASPP2 positive group (*n* = 29)		
	HK2 expression		*p*	HK2 expression		*p*	HK2 expression		*p*
	Negative (*n* = 40)	Positive (*n* = 40)		Negative (*n* = 21)	Positive (*n* = 30)		Negative (*n* = 19)	Positive (*n* = 10)	
Sex									
Male	34	32	0.556	17	23	0.57	17	9	1.0
Female	6	8		4	7		2	1	
Age (years)									
<50	21	17	0.37	14	14	0.158	7	3	1.0
≥50	18	23		7	16		12	7	
HBsAg									
Negative	4	3	1.0	1	3	0.876	3	0	0.532
Positive	36	37		20	27		16	10	
AFP (ng/mL)									
≤400	22	18	0.371	13	12	0.124	9	6	0.798
>400	18	22		8	18		10	4	
Tumour volume (cm^3^)									
≤5	23	20	0.501	15	12	0.027	8	8	0.119
>5	17	20		6	18		11	2	
Cirrhosis									
−	17	13	0.356	8	11	0.917	9	2	0.298
+	23	27		13	19		10	8	
AJCC Stage									
Stage I–II	11	17	0.16	5	11	0.33	6	6	0.28
Stage III–IV	29	23		16	19		13	4	
Recurrence time (months)									
≤6	13	15	0.639	7	7	0.431	6	8	0.037
>6	27	25		14	23		13	2	

*Note*: *p*‐values are two‐tailed and based on the Pearson χ^2^ test.

In univariate analysis, tumour size, ASPP2 and HK2 expression status were found to be the prognostic factors for RFS and OS (*p* < 0.05, Table 3). In multivariate analysis, tumour size, ASPP2 and HK2 expression status were shown as significant independent predictors of RFS and OS (Table 4). Patients with HK2‐high expression were 1.883 times more likely to suffer from relapse than patients with HK2‐low expression (hazard ratio, 1.883; 95% confidence interval, 1.094–3.243). Patients with ASPP2‐high expression were about 0.488 times less at risk of relapse than patients with ASPP2‐low expression (hazard ratio, 0.488; 95% confidence interval, 0.269–0.888). Therefore, increased expression of HK2 with decreased ASPP2 may serve as a prognostic indicator for patients with HCC.

**TABLE 3:** Univariate analyses of factors associated with recurrence‐free Survival (RFS) and overall survival (OS)

Variables	RFS		OS	
	Hazard ratio (95% CI)	*p*	Hazard Ratio (95% CI)	*P*
Gender (male vs. female)	0.771 (0.408–1.458)	0.424	0.76 (0.402–1.438)	0.399
Age, years (≥50 vs. < 50)	0.599 (0.356–1.006)	0.053	0.688 (0.410–1.154)	0.157
HbsAg (positive vs. negative)	1.398 (0.505–3.871)	0.519	1.192 (0.429–3.313)	0.736
AFP, ng/mL (>400 vs. ≤400)	1.508 (0.898–2.532)	0.12	1.333 (0.794–2.237)	0.277
Cirrhosis (yes vs. no)	1.251 (0.727–2.154)	0.418	1.136 (0.66–1.953)	0.646
Tumour size, cm (>5 vs. ≤ 5)	3.261 (1.812–5.869)	0.00	2.867 (1.601–5.133)	0.00
AJCC stage (III‐IV vs. I‐II)	1.602 (0.908–2.825)	0.104	1.5 (0.851–2.644)	0.161
ASPP2 (high vs. low)	0.468 (0.261–0.841)	0.011	0.435 (0.243–0.779)	0.005
HK2 (high vs. low)	1.74 (1.035–2.924)	0.037	1.841 (1.093–3.102)	0.022

**TABLE 4:** Multivariate analyses of factors associated with recurrence‐free Survival (RFS) and overall survival (OS)

	Hazard ratio (95% CI)	*p*
RFS		
Tumour size, cm (>5 vs. ≤5)	3.267 (1.804–5.915)	0.00
ASPP2 (high vs. low)	0.488 (0.269–0.888)	0.019
HK2 (high vs. low)	1.883 (1.094–3.243)	0.022
OS		
Tumour size, cm (>5 vs. ≤ 5)	2.849 (1.584–5.124)	0.00
ASPP2 (high vs. low)	0.53 (0.294–0.958)	0.036
HK2 (high vs. low)	1.802 (1.051–3.091)	0.032

*Note*: Multivariate analysis, cox proportional hazards regression model. Variables were adopted for their prognostic significance by univariate analysis and no obvious correlation between each other.

## 4 DISCUSSION

ASPP2 was first identified as a regulator of apoptosis by activating p53 to suppress tumour growth.^8^, ^9^ Subsequently, ASPP2 was found to inhibit tumour growth and metastasis through regulation of autophagy and epithelial plasticity independent of p53.^12^, ^13^, ^14^ Our recent data showed that ASPP2 inhibited tumour‐initiating capabilities and tumour growth by regulating mevalonate metabolism in HCC.^15^ Thus, deregulation of ASPP2 is involved in tumour metabolic reprogramming to benefit tumour growth and metastasis.

Here we showed that ASPP2 can regulate tumour‐initiating capability and promote tumour growth by inhibiting WNT/β‐catenin signalling mediated glycolysis metabolism in HCC. ASPP2‐depleted HCC cells exhibit higher glucose uptake, pyruvate and lactic acid production, increased cancer stemness characters and tumour growth. Moreover, this increased tumour growth and stemness characteristics could be reversed using inhibitors of glycolysis metabolism pathway and β‐catenin signalling pathway.

Previous studies have shown that ASPP2 binds the β‐catenin‐E‐cadherin complex to regulate epithelial plasticity, and decreased‐ASPP2 in HCC induces β‐catenin entrance into the nucleus and activation of its target genes.^13^ One major driver of liver cancer independent of aetiology is the activated Wnt–β‐catenin pathway.^17^ Wnt/β‐catenin signalling plays an important role in tumour growth as well as in metabolic reprogramming.^18^ Activated Wnt/β‐catenin signalling pathways shift cancer metabolism from mitochondrial oxidative phosphorylation to Warburg glycolytic metabolism.^19^, ^20^, ^21^ When the WNT pathway is activated in cancers, the number of β‐catenin in the nucleus increases significantly and leads to the expression of many downstream genes, such as PDK1 and c‐myc,^22^ to promote glycolysis.^16^

In our study, immunoblotting analysis showed that ASPP2 could significantly inhibit β‐catenin translocation into the nucleus, and the elevated levels of mRNA and protein of HK2、PKM2 induced by ASPP2 knockdown were inhibited by WNT/β‐catenin signalling inhibitors XAV‐939 and ICG‐001. These data suggest that ASPP2 may inhibit glycolysis kinase expression via suppressing WNT/β‐catenin signalling. Our results revealed that XAV‐939 and ICG‐001 attenuated the elevated levels of ECAR and extracellular lactate induced by ASPP2 knockdown, suggesting that ASPP2 inhibits aerobic glycolysis of HCC by inhibiting glycolysis kinase expression via the WNT/β‐catenin signalling.

In the examination of 80 cases of HCC tissue, the expression of ASPP2 and HK2 was significantly inversely correlated. Patients with ASPP2‐high and HK2‐low expression exhibited the best recurrence‐free survival and overall survival. These data further demonstrate that ASPP2‐regulated Warburg effect contributes to tumour progression in HCC patients.

In summary, we investigated the roles and mechanisms of ASPP2 in the glycolysis metabolism of HCC. Downregulation of ASPP2 activates the WNT/β‐catenin pathway contributing to enhanced glycolysis kinase expression, and leading to the promotion of HCC proliferation, glycolysis metabolism, stemness and drug resistance. This ASPP2‐induced inhibition of glycolysis metabolism is WNT/β‐catenin pathway dependent. Altogether our findings elucidate new insights into the roles of ASPP2 act on glycolysis pathway and suggest that may be promising as a new therapeutic strategy for HCC treatment.

## AUTHOR CONTRIBUTIONS

**beibei liang:** Investigation (equal); methodology (equal); writing – original draft (equal). **Yuan Jiang:** Data curation (equal); investigation (equal); methodology (equal); writing – original draft (equal). **Shaohua Song:** Resources (equal); supervision (equal). **Wei Jing:** Resources (equal). **Hao Yang:** Data curation (equal); methodology (equal). **Li Zhao:** Resources (equal). **Ya Chen:** Investigation (equal). **Qiqi Tang:** Investigation (equal); software (equal). **Xuhui Li:** Methodology (equal). **Lisha Zhang:** Methodology (equal). **Haili Bao:** Resources (equal). **Gang Huang:** Conceptualization (equal); funding acquisition (equal). **Jian Zhao:** Conceptualization (equal); funding acquisition (equal); project administration (equal); writing – review and editing (equal).

## CONFLICT OF INTEREST STATEMENT

The authors declare no conflicts of interest.

## Supporting information


Figure S1.

Figure S2.

Figure S3.

Table S1.

Table S2.

Appendix S1.

## Data Availability

All remaining data are available within the article and supplementary files, or available from the authors upon request.
