# Supplementary figures and images for: ASPP2 suppresses tumour growth and stemness characteristics in HCC by inhibiting Warburg effect via WNT/β‐catenin/HK2 axis
Source: J Cell Mol Med. 2023 Feb 8;27(5):659–71. doi: 10.1111/jcmm.17687 (PMC9983321; doi:10.1111/jcmm.17687)

(A)

HCC-LM3

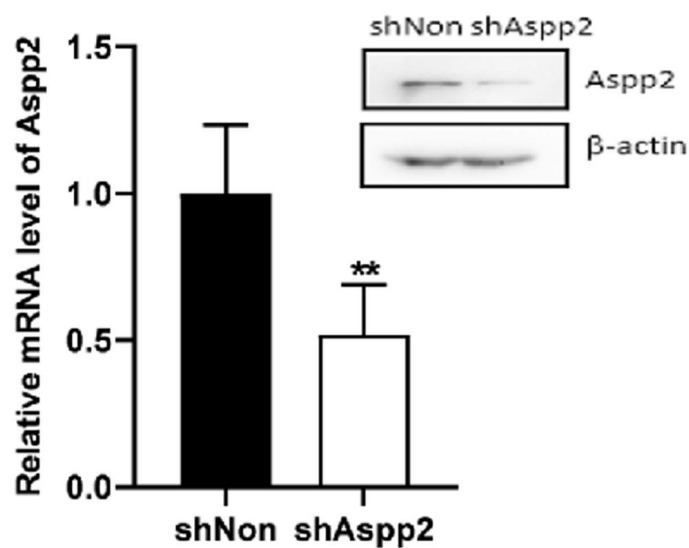

(B)

Hep-G2

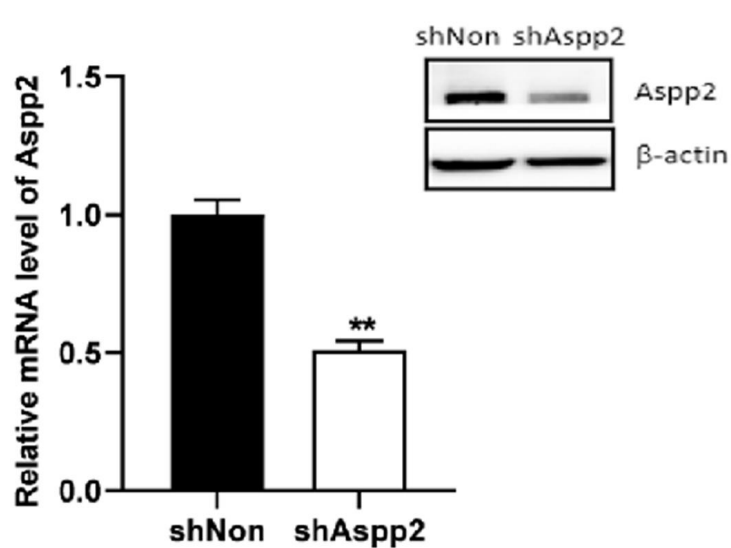

(C)

Hep-3B

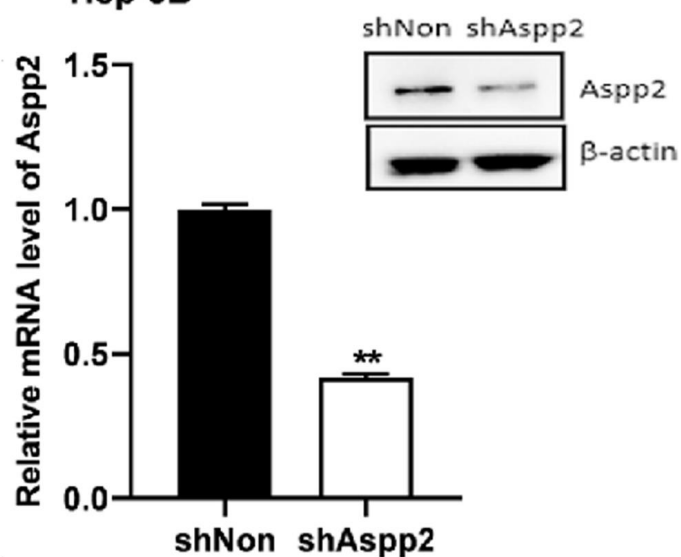

(D)

Huh-7

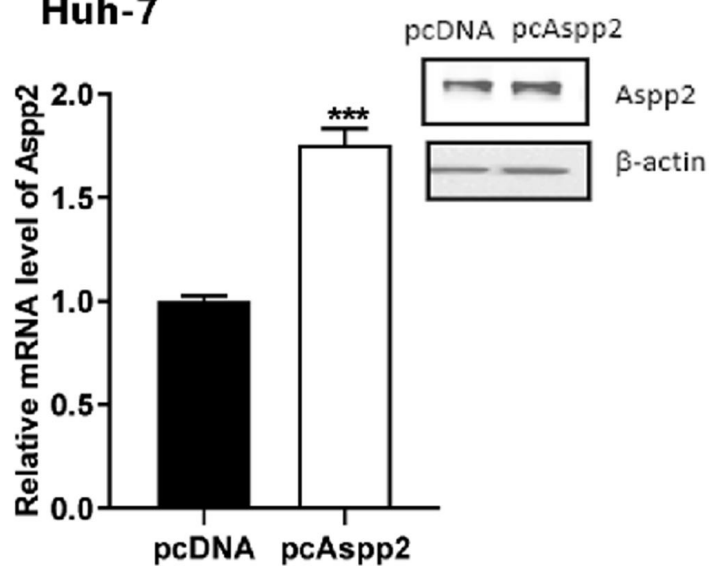

Supplement: Supplementary file 1 — Figure S1. [file JCMM-27-659-s006.pdf]

(A)

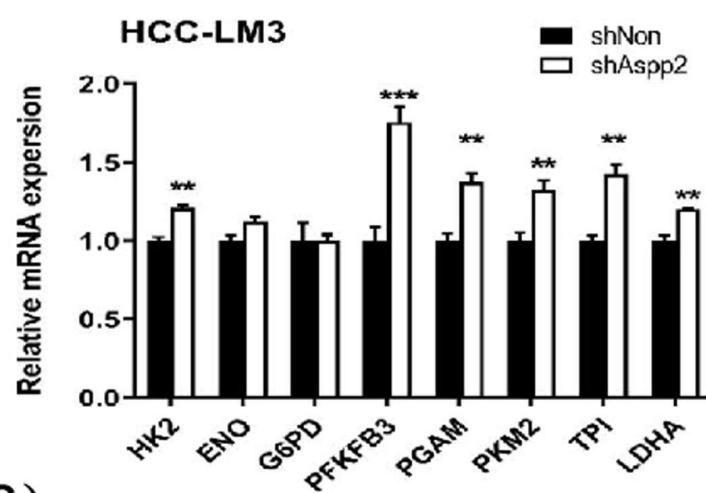

(B)

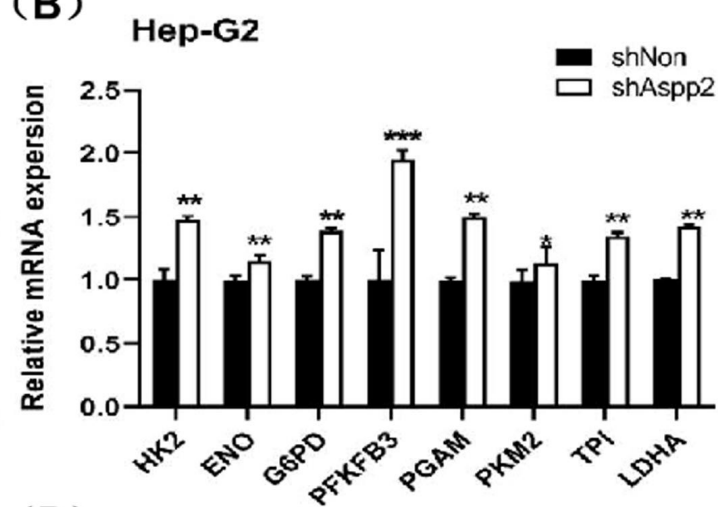

(C)

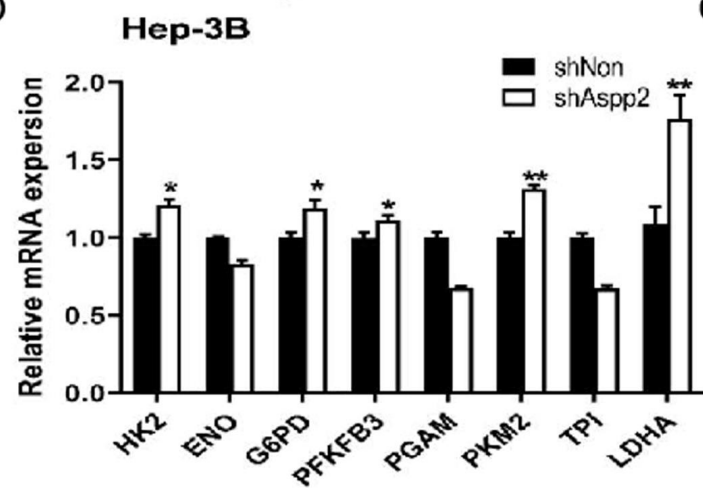

(D)

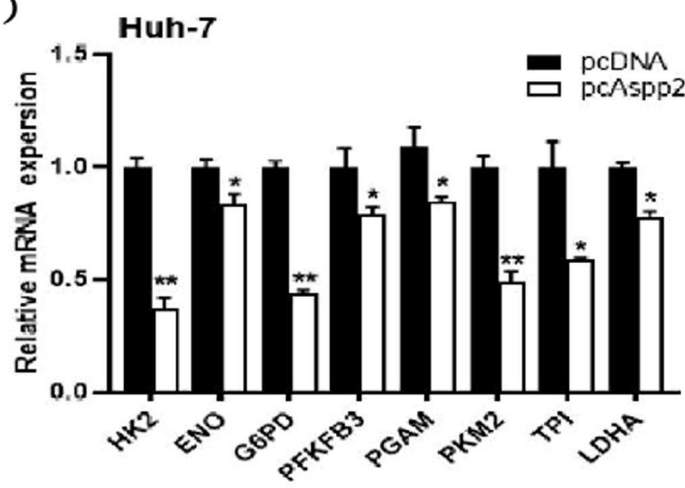

Supplement: Supplementary file 2 — Figure S2. [file JCMM-27-659-s005.pdf]

(A)

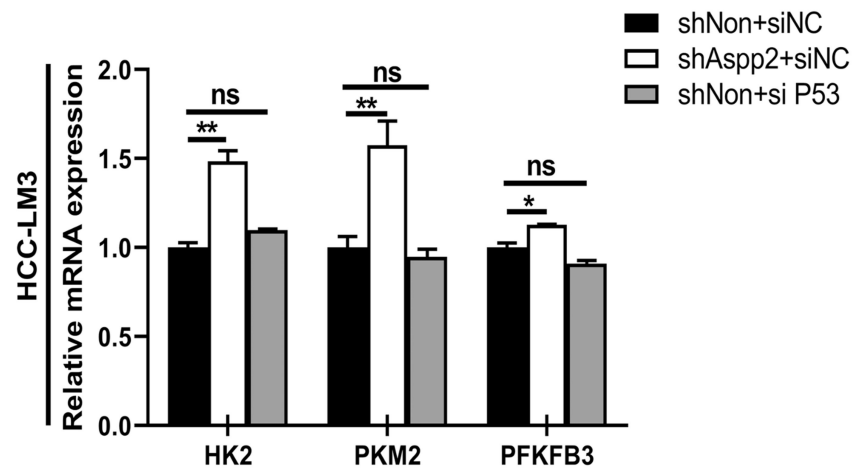

(B)

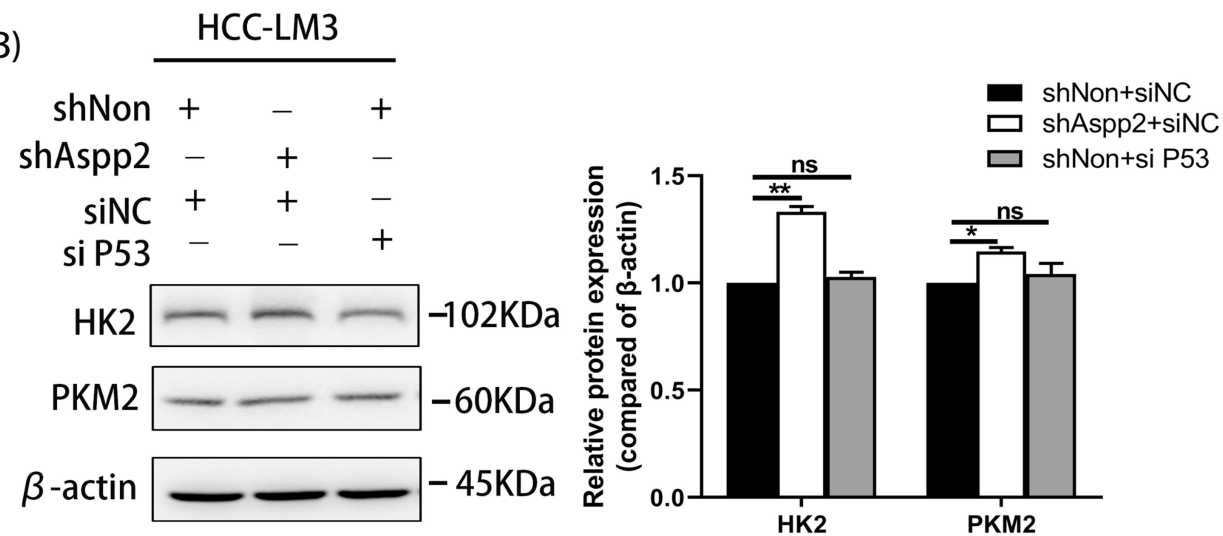

(C)

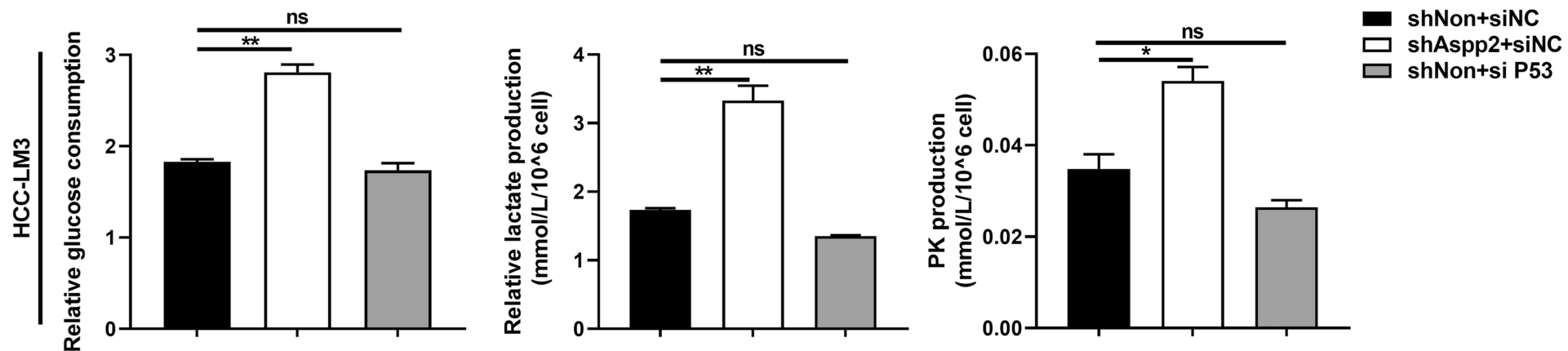

Supplement: Supplementary file 3 — Figure S3. [file JCMM-27-659-s002.pdf]
