# Supplementary material for: ASPP2 suppresses tumour growth and stemness characteristics in HCC by inhibiting Warburg effect via WNT/β‐catenin/HK2 axis
Source: J Cell Mol Med. 2023 Feb 8;27(5):659–71. doi: 10.1111/jcmm.17687 (PMC9983321; doi:10.1111/jcmm.17687)
Supplement: Supplementary file 4 — Table S1. [file JCMM-27-659-s004.doc]

**Table S1. Antibodies used in this study**

| **Protein** | **Usage** | **Antibody Dilution** |
| --- | --- | --- |
| ASPP2 | WB | A4480, Sigma-Aldrich 1:1000 |
| ASPP2 | IHC | A4480, Sigma-Aldrich 1:50 |
| V5 | WB | V8137, Sigma-Aldrich 1:1000 |
| α-tubulin | WB | 11224-1-AP, Proteintech 1:1000 |
| Lamin B | WB | 12987-1-AP, Proteintech 1:1000 |
| β-catenin | WB | 8480, Cell signal technology 1:1000 |
| PKM2 | WB | 4053, Cell signal technology 1:1000 |
| PKM2 | IHC | 4053, Cell signal technology 1:100 |
| Hexokinase 2 | WB | 22029-1-AP ,Proteintech 1:1000 |
| Hexokinase 2 | IHC | 22029-1-AP , Proteintech 1:50 |
| C-myc | WB | 9402,Cell signal technology 1:1000 |
| CyclinD1 | WB | 2922,Cell signal technology 1:1000 |
| P21 | WB | 2947,Cell signal technology 1:1000 |
| PFKFB3 | WB | ab181861, abcam 1:1000 |

**Note:** Secondary antibodies used in Western blot analysis, goat anti-rabbit IgG-HRP (sc-2030), goat anti-mouse IgG-HRP(sc-2031) and donkey anti-goat IgG-HRP (sc-2020) were purchased from Santa Cruz Biotechnology. Abbreviations: WB, Western blotting; IF, immunofluorescence; IHC, immunohistochemistry.
