# Supplementary material for: ASPP2 suppresses tumour growth and stemness characteristics in HCC by inhibiting Warburg effect via WNT/β‐catenin/HK2 axis
Source: J Cell Mol Med. 2023 Feb 8;27(5):659–71. doi: 10.1111/jcmm.17687 (PMC9983321; doi:10.1111/jcmm.17687)
Supplement: Supplementary file 5 — Table S2. [file JCMM-27-659-s001.doc]

**Table S2. Primers used in this study**

| **Primers for Real-time PCR** | | |
| --- | --- | --- |
| **Protein** | | **Sequence (5’→3’)** |
| ASPP2  ABCG2 | F  R  F  R | GAAGACTCGGTGAGCATGCG  GCGATACGCTCTGAGCCAGT  CAGGTGGAGGCAAATCTTCGT  ACCCTGTTAATCCGTTCGTTTT |
| Oct4 | F | CGCAAGCCCTCATTTCAC |
|  | R | CATCACCTCCACCACCTG |
| EpCAM  CD44 | F  R  F  R | AATCGTCAATGCCAGTGTACTT  TCTCATCGCAGTCAGGATCATAA  CTGCCGCTTTGCAGGTGTA  CATTGTGGGCAAGGTGCTATT |
| CB | F | AGATGTAGGCCGGGTGATCT |
|  | R | CCGCCCTGGATCATGAAGTC |
| HK2 | F | AGCCCTTTCTCCATCTCCTT |
|  | R | GCTTGCCTACTTCTTCACGG |
| PKM2  PFKFB3 | F  R  F  R | ATGTCGAAGCCCCATAGTGAA  TGGGTGGTGAATCAATGTCCA  TGAAATGTCCGCTCCACACT  AGCTCTTCATGTTCTCTGACC |
| LDHA | F  R | AACTTGGCGCTCTACTTGCT  GGACTTTGAATCTTTTGAGACCTT |
