# Supplementary material for: ASPP2 suppresses tumour growth and stemness characteristics in HCC by inhibiting Warburg effect via WNT/β‐catenin/HK2 axis
Source: J Cell Mol Med. 2023 Feb 8;27(5):659–71. doi: 10.1111/jcmm.17687 (PMC9983321; doi:10.1111/jcmm.17687)
Supplement: Supplementary file 6 — Appendix S1. [file JCMM-27-659-s003.docx]

**Supplementary Material**

**1.Materials and methods**

**1.1 Western blotting.**

Total protein was extracted from cell samples with Cell lysis buffer for Western and IP (Beyotime, Shanghai) with 1% PMSF (Beyotime, Shanghai) .The protein concentrations were measured using the Bicinchoninic Acid (BCA) kit (YEASEN, Shanghai). Proteins were separated by SDS-PAGE and transferred to PVDF membrane (Millipore, USA). Then the blots were detected with super ECL Detection reagent (YEASEN, Shanghai) using Bio-Rad ChemiDocTM (Azure Biosystems, USA) after incubated with primary antibodies overnight at 4℃ and probed with secondary antibodies at room temperature for 1-2 h. ASPP2 antibody (A4480) used at 1:1000 was purchased from Sigma-Aldrich. PKM2 (4053),β-catenin (8480) antibodies used at 1:1000 were purchased from Cell signal teleology. HK2 (22029-1-AP) , Lamin B（12987-1-AP）and α-Tubulin (11224-1-AP) antibodies used at 1:100-1:1000 were purchased from proteintech. XAV-939（HY-15147），ICG-001（HY-14428）were all purchased from MedChemExpress (USA), 2-DG(SIGMA,D8375) , 3-bp(S5426） was purchased from Selleck.

**1.2 shRNA design and construction**

We designed three pairs of cDNA oligonucleotides targeting ASPP2 mRNA expression, using web-based software from Invitrogen (http://rnaidesigner.invitro gen. com/rnaiexpress/) and InvivoGen Inc. (San Diego,CA; http:// www. sirnawizard. com/design. php) . After synthesis, we inserted these double-strand oligos into the vector pENTR/U6 (Invitrogen) and sequenced the resulting plasmids to ensure the shRNA construct targeted human ASPP2 expression or were scrambled,which were generated and designated as LV-shAspp2 and LV-shNon. Then, the plasmids were transfected into HCC-LM3 cells and gene silencing efficiency was validated 72 hours after transfection by Real-time PCR and Western blot The double-strand oligo DNAs with the silencing efficiency for ASPP2 were as follows: 5’-GCTGAGGGA GAAAGAGAAGAA-3’; We used scramble shRNA (5’-AATTCTCCGAACGTG TCACGT-3’) as a negative control.

**1.3 Small interfering RNA construction.**

Three pairs of siRNAs targeting P53 were designed by GenePharma (Shanghai, China). The sequences were used as follows: for P53-homo-278 (siP53), sense 5’ -CCCGGACGAUAUUGAACAATT -3’ and antisense 5’ -UUGUUCAAUAUCGU CCGGGTT -3’.

**1.4 Measurement of Glucose Uptake**

The cell culture medium was collected in each experiment. The glucose concentration in the medium was measured using a Glucose Assay Kit (GAG020, Sigma-Aldrich) according to the manufacturer's instructions, with using a 96-well plate. Briefly, 1µL of diluted (100X) sample was mixed with 100 µL of the assay reagent and incubated for 30 min at 37°C. The colorimetric reaction was stopped with 100 µL of 6M H_2_SO_4._ The absorbance was measured at a wavelength of 540 nm using a microplate reader (A51119500C，Thermo Scientific™). The glucose concentration of the medium was calculated based on the standard curve in each experiment and normalized with cell number.

**1.5 Lactate /****pyruvic production**

Lactate levels in the culture media of cells were determined by using a Lactate Assay Kit (A019-2-1,Jiancheng Bioengineering ). The pyruvic level in the cells was measured using a Pyruvic Assay kit (BC0549,Solarbio). Cells (1×10^6^) were seeded onto 6-well plates for 12h and then incubated 10 μM XAV-939, or 10 μM ICG-001 for 24h. Cells were then incubated for 12h and the culture medium was collected for measurement of lactate concentrations, the cells were collected for measurement of the pyruvic production . The absorbance of lactate production was measured at a wavelength of 530 nm using a microplate reader (A51119500C，Thermo Scientific™) and the pyruvic production was measured at a wavelength of 340 nm. Calculate the data according to the formula and normalized with cell number.

**1.6 Tumor xenograft model.**

Male athymic BALB/c nude mice aged 4 weeks were purchased from Shanghai Experimental Animal Center of Chinese Academic of Sciences (Shanghai, China). We had four groups,five in each group. Animal were assigned randomly to experimental or control groups by blind selection.All animals were kept and bred in a controlled environment and housed in a pathogen-free barrier facility with a 12h light/dark cycle under a temperature of 21°C and a relative humidity of 50% according to the guidelines of Shanghai Medical Experimental Animal Commission. Animal experiments were approved by Medical Ethics Committee of Shanghai University of Medicine & Health Sciences( 2018-GZR-18-310110196803058627). After infection with shNon-Luc and shAspp2-Luc (at a MOI of 50), HCC-LM3 cells (1 × 10^7^) were implanted into the flank of nude mice by subcutaneous injection (male BALB/c nu/nu, five in each group). Tumors were well-established after 7 days. 2-DG (500 mg/kg body weight) in 0.9% isotonic saline was given to mice daily for 3 days by [intraperitoneal injection](SCI文件/AppData/Local/youdao/dict/Application/7.5.2.0/resultui/dict/result.html%3fkeyword=intraperitoneal injection&lang=en) for 4 weeks. Then the mice were sacrificed and the tumor tissues were isolated for histopathology experiments.

**1.7 ^18^F-FDG PET/CT Imaging**

^18^F-FDG PET/CT was performed using a PET/ CT scanner (Mediso, Hungary; Bioscan, USA), whole-body PET/CT was scanned 30 min after the intravenous injection of 100 μCi/kg of ^18^F-FDG. To evaluate ^18^F-FDG accumulation, Standardized Uptake Value (SUVmean) was measured.

**1.8 Patient samples.**

We performed a tissue microarray constructed by Shanghai Weiao Biotechnology Co., Ltd, China (Weiao Biotechnology Co.,ZL-LVC1605). Weiao Biotechnology Co. was responsible for obtaining informed consent from all subjects. Eighty primary HCC samples made for microarray were obtained from patients who had undergone curative hepatic resection between 2008 and 2015. Patient’s samples were approved to use for research purposes by medical Ethics Committee of Shanghai University of Medicine & Health Sciences( 2018-GZR-18- 310110196803058627). We defined curative resection as complete resection of all tumor nodules and the cut surface being free of cancer by histologic examination. The clinicopathologic features of the patients were summarized in table2. Patient follow-up was completed on December 15, 2015. The median follow-up period was 47 months (range, 1–80 months). Overall survival (OS) was defined as the interval between the dates of surgery and death. Recurrence-free survival (RFS) was defined as the interval between the dates of surgery and recurrence, Patients were censored on the date of death or the last follow-up if recurrence was not diagnosed.

**1.9 Immunohistochemical staining**.

The expressions of ASPP2, HK2 and PKM2 were analyzed with the ImageScope system in formalin-fixed, paraffin-embedded sections of primary tumors. Briefly, the slides were dewaxed, hydrated ,quenched endogenous peroxidase activity, retrieved antigen, blocked and incubated with the antibody against ASPP2 (1:50, A4480,Sigma-Aldrich), HK2 (1:50, 22029-1-AP, Proteintech) or PKM2 (1:100, 4053,Cell signal technology)overnight at 4°C. Then, sections were rinsed and incubated with the working solution of horseradish peroxidase-labeled goat anti-rabbit for 1h at 37°C. After rinse for three times, diaminobenzidine colorimetric reagent solution from Dako (Carpinteria, CA) was used. Subsequently the slides were counterstained by hematoxylin and dehydrated in graded alcohol and mounted. Evaluation of immunostaining was independently performed by two experienced pathologists. The expression of ASPP2 and HK2 were scored according to the signal intensity and distribution. Briefly, a mean percentage of high tumor cells were determined in at least five areas at ×400 magnification and assigned to one of the five following categories: 0, <5%; 1, 5-25%; 2, 25-50%; 3, 50-75% and 4, >75%. The intensity of immunostaining was scored as follows: 1, weak; 2, moderate and 3, intense. For tumors that showed heterogeneous staining, the predominant pattern was taken into account for scoring. The staining intensity and the percentage of high tumor cells were multiplied to generate a weighted score for each case. Tissues with immunohistochemical scoring≤2 were considered as low, 3-12 as high.

1. **Abbreviations:**

ASPP2, the Ankyrin-repeat-containing, SH3-domain-containing and Proline-rich region-containing Protein 2; HCC, hepatocellular carcinoma; 5-FU,Fluorouracil

qRT-PCR, Quantitative real-time PCR; GC-MS, Gas chromatography–mass spectrometry; TCGA, The Cancer Genome Atlas ; RFS,recurrence-free survival;

OS,overall survival; ECAR, Extracellular acidification rate; OCR,oxygen consumption rate ; 2-DG, 2-Deoxy-D-glucose ; 3-bp, 3-Bromopyruvic acid;

HK2, hexokinase 2 ; PFKFB3,6-phosphofructo-2-kinase/fructose-2, 6-bisphosphatase 3; PKM2, pyruvate kinase M2; AFP, alpha-fetoprotein; AJCC, American Joint Committee on Cancer

1. **Supplementary Figure Legends ：**

**Figure S1. Effect of silencing and overexpression of ASPP2 in HCC cells**. The ASPP2 protein evaluation after silencing and overexpression of ASPP2 in HCC cells. HCC-LM3, HepG2 and Hep-3B cells(A-C) were infected with LV-shNon or LV-shAspp2 for 72 hours. Huh-7 cells(D) were transfected with pcDNA3.0 or pcAspp2 for 48 hours. The expression of ASPP2 was detected by qRT-PCR and western blot. The same bands of β-actin in Hep-3B cells are used in figure2B and figure S1C .And,the same bands of β-actin in Huh-7 cells are used in figure2 B and and figure S1D . Data represented the mean ± SD *, *P* < 0.05; **, *P* < 0.01, ***, *P* < 0.001

**Figure S2. ASPP2 effects the expression of key emzymes of the Warburg effect in HCC cells.** HCC-LM3, HepG2 and Hep-3B cells(A-C) were infected with LV-shNon or LV-shAspp2 for 72 hours. Huh-7 cells(D) were transfected with pcDNA3.0 or pcAspp2 for 48 hours. The mRNA expression of glycolytic targeted genes were detected by qRT-PCR. Data represented the mean ± SD *, *P* < 0.05; **, *P* < 0.01, ***, *P* < 0.001

**Figure S3.** ASPP2 may regulate aerobic glycolysis in a p53-independent manner (A) The mRNA levels of HK2, PFKFB3 and PKM2 were determined after ASPP2 knowdown or P53 inhibition in HCC-LM3 cells. (B) The protein levels of HK2 and PKM2 were determined after ASPP2 knockdown or P53 inhibition in HCC-LM3 . (C)Pyruvic acid production, lactate production, and glucose consumption in HCC-LM3 infected with LV-shAspp2 and LV-shNon or treated with siP53 All data are shown as the mean ± SEM of 3 independent experiments. * *P*< 0.05; ***P*< 0.01 ns:no [significance](javascript:;).
